# Effect of Different Application Modalities on the Bonding Performance of Adhesive Systems to Dentin: A Systematic Review and Meta-Analysis

**DOI:** 10.3390/cells12010190

**Published:** 2023-01-03

**Authors:** Louis Hardan, Rim Bourgi, Carlos Enrique Cuevas-Suárez, Walter Devoto, Maciej Zarow, Paulo Monteiro, Natalia Jakubowicz, Amine El Zoghbi, Dariusz Skaba, Davide Mancino, Naji Kharouf, Youssef Haïkel, Monika Lukomska-Szymanska

**Affiliations:** 1 Department of Restorative Dentistry, School of Dentistry, Saint-Joseph University, Beirut 1107 2180, Lebanon; 2 Department of Biomaterials and Bioengineering, INSE&RM UMR_S 1121, Biomaterials and Bioengineering, 67000 Strasbourg, France; 3 Dental Materials Laboratory, Academic Area of Dentistry, Autonomous University of Hidalgo State, Circuito Ex Hacienda La Concepción S/N, San Agustín Tlaxiaca 42160, Hidalgo, Mexico; 4 Independent Researcher, 16030 Liguria, Italy; 5 “NZOZ SPS Dentist” Dental Clinic and Postgraduate Course Centre, pl. Inwalidow 7/5, 30-033 Cracow, Poland; 6 Clinical Research Unit (CRU), Centro de Investigação Interdisciplinar Egas Moniz (CiiEM), Egas Moniz, CRL, Monte de Caparica, 2829-511 Caparica, Portugal; 7 Department of Prosthetic Dentistry and Occlusion, School of Dentistry, Saint-Joseph University, Beirut 1107 2180, Lebanon; 8 Department of Periodontal Diseases and Oral Mucosa Diseases, Faculty of Medical Sciences in Zabrze, Medical University of Silesia, 40-055 Katowice, Poland; 9 Department of Endodontics, Faculty of Dental Medicine, Strasbourg University, 67000 Strasbourg, France; 10 Pôle de Médecine et Chrirugie Bucco-Dentaire, Hôpital Civil, Hôpitaux Universitaires de Strasbourg, 67000 Strasbourg, France; 11 Department of General Dentistry, Medical University of Lodz, 251 Pomorska St., 92-213 Lodz, Poland

**Keywords:** adhesion, dental adhesive, dental bonding, dentin bonding agent

## Abstract

Diverse types of dental adhesives exhibit different cytotoxic outcomes on cells in vitro. Currently, no standard adhesive application technique has so far been decisive for clinicians for better durability of resin–dentin bonds of adhesive systems. The purpose of this study was to systematically review the literature to evaluate the bonding performance of adhesive systems to dentin by using different application modalities. The systematic research strategy was conducted by two reviewers among multiple databases: PubMed, Scopus, Web of Science, Embase, and Scielo. In vitro studies reporting the effects of additional steps for the application of adhesive systems on the bond strength to dentin were selected. Meta-analysis was performed using Review Manager Software version 5.3.5 using the random effects model. The methodological quality of each in vitro study was assessed according to the parameters of a previous systematic review. The electronic research through different databases generated a total of 8318 references. After the examination of titles and abstracts, a total of 106 potentially relevant studies accessed the full-text evaluation phase. After full-text examination, 78 publications were included for the qualitative analysis, and 68 studies were included in the meta-analysis. Regarding the etch-and-rinse adhesive systems, the application modalities that improved the overall bond strength were the application of a hydrophobic resin layer (*p* = 0.005), an extended application time (*p* < 0.001), an application assisted by an electric current (*p* < 0.001), a double-layer application (*p* = 0.05), the agitation technique (*p* = 0.02), and the active application of the adhesive (*p* < 0.001). For self-etch adhesive systems, the techniques that improved the overall bond strength were the application of a hydrophobic resin layer (*p* < 0.001), an extended application time (*p* = 0.001), an application assisted by an electric current (*p* < 0.001), a double-layer application (*p* < 0.001), the agitation technique (*p* = 0.01), and the active application of the adhesive (*p* < 0.001). The in vitro evidence suggests that the application of adhesive systems using alternative techniques or additional strategies may be beneficial for improving their bond strength to dentin. The application modalities that favored the overall bond strength to dentin were an extended application time, a double-layer application, an application assisted by an electric current, the active application of the adhesive, and the application of a hydrophobic resin layer. Worth mentioning is that some techniques are intended to increase the degree of the conversion of the materials, and therefore, improvements in the biocompatibility of the materials can be expected.

## 1. Introduction

Over the years, adhesive systems have been introduced into many fields of modern restorative dentistry; however, there are still some unresolved problems concerning the durability of the resin–dentin bond interface [1]. Adhesion to enamel structures has become a predictable and well-established procedure [2], whereas adhesion to dentin, due to its heterogeneous structure and histology, has been considered defiant [3,4]. In fact, achieving an ideal interdiffusion of the adhesive system within collagen fibrils and the stability of the resin–dentin interface are of key importance [5]. Accordingly, it is fundamental to recognize the mechanism of dentin hybridization, in which an interdiffusion zone, also called the hybrid layer (HL), is created and, consequently, leads to the formation of micromechanical retention of the dental composite restoration [6]. Thus, the HL is a combination of residual hydroxyapatite (HAp), collagen, solvents, and resin monomers, and its strength ultimately relies on the resistance of each constituent to degradation phenomena [7,8]. 

Through adhesion procedures, the mineral component is partly or completely removed by the acidic monomers of self-etch (SE) or etch-and-rinse (E&R) adhesive systems [9]. A simplification of the conventional view of dental bonding by means of a faster application, a less-sensitive method, and numerous optional applicability is feasible today with the advent of universal adhesives [3,6,9]. These strategies have been used for enamel and dentin bonding to resin-based materials with a number of steps. Strengths have been focused regarding the lessening of the number of steps, the decrease in potential errors, and the reduction in the technique sensitivity related to the bonding procedure [9]. Currently, these dentin bonding agents are comprised of monomers with both hydrophobic and hydrophilic groups, polymerization modulators, and somewhat high concentrations of organic solvents [10]. These solvents help as diluting agents and enhance the spreading, wetting, and penetration of monomers within the microporosities of the exposed, acid- demineralized collagen network [11]. However, it is important to emphasize that the higher the solvent content within the polymer, the lower the resin–dentin bond strength and the mechanical properties of the cured resin [12].

Ideally, any remaining solvent must be evaporated from the dentinal surface by air-drying the applied adhesive prior to photoactivation. The presence of a residual solvent might jeopardize the polymerization of resin monomers, affecting the integrity of the bond, compromising the quality of the polymer inside the HL, and producing undesirable voids within the adhesive interface. These voids may act as defect-initiator sites, providing a pathway for nanoleakage, causing a decrease in bond strength and mechanical properties [13,14]. Solvent evaporation can be achieved by allowing an evaporation time between the application and the curing of the adhesive or by air-drying using an air syringe [15]. All in all, the evaporation of solvents from the adhesive depends on numerous features, such as the type of solvent, the tooth–syringe distance, the type of monomer, operator skills, and the air temperature, which appears to impact the air-drying time of an adhesive system [16].

Furthermore, there are different strategies that can be used to facilitate the removal of solvents and enhance the durability of adhesive systems, such as prolonged air-drying, using a warm-air stream on the primer or the adhesive system [7], a prolonged application time, active bonding application, multiple adhesive coatings, an extra layer of hydrophobic coat, and increased light-curing exposure [17]. 

Suitably, a better definition of the gold standard technique for applying adhesive systems to dentin should be of great consideration. Accordingly, many approaches, including active bonding application, multiple-layer application (two layers or more), and the modification of the adhesive application time, might be introduced by clinicians in their daily practice in order to improve the bond strength of adhesive systems to dentin. The issue is that, until now, there has been no ideal protocol for achieving the stable and optimal adhesion of adhesive systems to dentin. Likewise, the improvement in bond strength can be realized by means of several strategies and, thus, has been advocated by various authors [5,7,9,10,17,18].

Another topic to be addressed is related to the fact that as these materials come in close and prolonged contact with vital dentin, biocompatibility is one of the most critical requirements for dental adhesives [19]. Methacrylate monomers (i.e., bis-GMA, UDMA, and HEMA) were reported to induce toxicity via GSH (intracellular glutathione) depletion, cell cycle arrest, apoptosis/necrosis, and even apoptosis of human gingival fibroblast [20,21]. It is a well-known fact that morphological changes in several types of cells, apoptosis, and growth suppression can be generated by HEMA [22,23]. The release of resin monomers has been proposed as one of the possible causes of the adverse effects of dentin adhesives [24], and therefore, the definition of the gold standard technique for applying adhesive systems to dentin must imply a technique that ensures the achievement of the highest possible degree of conversion of the material.

Apart from the above report and to the extent of the researchers’ knowledge, no standard adhesive application technique has so far been decisive for clinicians for better durability of resin–dentin bonds of adhesive systems. Therefore, the purpose of this study is to systematically review the literature to evaluate the bonding performance of adhesive systems to dentin by using different application modalities. The null hypothesis to be tested is that different application modalities do not provide similar bond strength to dentin when compared to the application of the adhesive, according to the manufacturer’s instructions.

## 2. Materials and Methods

The present systematic review and meta-analysis was conducted following the guidance of PRISMA (preferred reporting items of systematic reviews and meta-analyses) in order to follow a uniform and transparent methodology [25]. The following PICOS framework was used: population, dentin; intervention, different application modalities; control, application of the adhesive according to the manufacturer’s instructions; outcomes, bond strength; and study design, in vitro studies. The research question was: “Does the use of different application modalities improve the bonding performance of adhesive systems to dentin?”

### 2.1. Literature Search

The systematic research strategy was conducted by two (R.B. and C.E.C.-S.) reviewers among multiple databases: PubMed, Scopus, Web of Science, Embase, and Scielo. The literature search was performed on 2 August 2022. The search strategy used in PubMed is described in the Table 1. The search strategy for the other databases was adapted from that used for PubMed. After the search, all articles were imported into Mendeley Desktop 1.17.11 software (Glyph & Cog, LLC, London, UK) to eliminate duplicates. 

### 2.2. Study Selection

All the articles were imported into Rayyan online tool [26], and the titles and abstracts were initially screened to identify studies that potentially met the following eligibility criteria: (1) in vitro studies reporting the effect of the use of additional steps for the application of adhesive systems on the bond strength to dentin; (2) evaluating the bond strength of adhesive systems to dentin with a resin-based material as an antagonist; (3) including a control group in which the adhesive system was applied according to manufacturers’ instructions; (4) including mean and standard deviation (SD) data in MPa on shear, micro-shear, tensile, and micro-tensile bond tests. Only manuscripts published in the English language were considered. Case series, case reports, pilot studies, and reviews were excluded. Afterwards, full texts were reviewed, and a systematic methodology was used to label all the relevant information for the exclusion or the inclusion of the individual papers. The decision process was performed by two independent reviewers (L.H. and R.B.). In case of disagreement between the reviewers, the final decision was reached through consultation with a third reviewer (C.E.C.-S.), a senior, experienced researcher.

### 2.3. Data Extraction

The results of the selected studies were extracted using Microsoft Office Excel 2019 (Microsoft Corporation, Redmond, WA, USA). These data comprised the year of publication, the type of adhesive system, strategy evaluated for bonding to dentin, outcomes evaluated, the type of bond strength test evaluated, and storage conditions. Additionally, the mean, SD, and number of specimens from the bond strength test were collected, and these data were then subjected to meta-analysis.

### 2.4. Quality Assessment

The methodological quality of each in vitro study was assessed by two reviewers (L.H. and R.B.), according to the parameters of a previous systematic review [17]. The risk of bias in each article was evaluated according to the description of the following parameters: specimen randomization, single operator, operator blinded, control group, standardized specimens, failure mode, manufacturers’ instructions, and sample size calculation. If the authors reported the parameter, the study received a “YES” for that specific parameter. In the case of missing information, the parameter received a “NO.” The risk of bias was classified according to the sum of “YES” answers received: 1 to 3 indicated a high bias, 4 to 6 medium, and 7 to 8 indicated a low risk of bias.

### 2.5. Statistical Analysis

Meta-analysis was performed using Review Manager Software version 5.3.5 (The Nordic Cochrane Centre, The Cochrane Collaboration, Copenhagen, Denmark). The analysis was carried out using the random-effects model, and pooled-effect estimates were obtained by comparing the standardized mean difference between bond strength values obtained from the control and experimental groups. The control group was considered when the adhesive system was applied according to the manufacturers’ instructions, while the experimental group was considered when the adhesive system was applied using a different approach. In studies where several experimental groups were compared with the same control group, data from the experimental groups (mean, SD, and sample size) were combined for the meta-analysis [27]. E&R and SE adhesives were analyzed separately. Subgroups considering the immediate and long-term bond strength data were used. A *p*-value < 0.05 was considered statistically significant. Statistical heterogeneity of the treatment effect among studies was assessed using the Cochran Q test and the inconsistency I^2^ test.

## 3. Results

**Figure 1:**
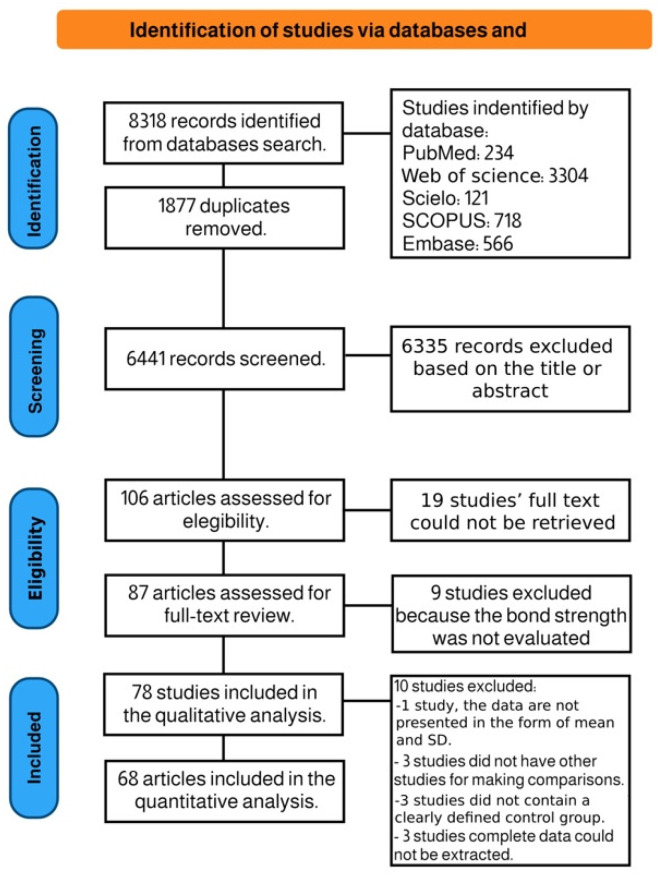
Prisma flowchart according to PRISMA guidelines.

The electronic research through three different databases generated a total of 8318 references, which were reduced to 6441 after duplicate removal. After the examination of titles and abstracts, 6335 studies were excluded because of their study design incompatibility with this review. A total of 106 potentially relevant studies accessed the full-text evaluation phase. From these, 19 were excluded because there was no access to the full-text manuscript [28,29,30,31,32,33,34,35,36,37,38,39,40,41,42,43,44,45], resulting in a final number of 87 publications included for further qualitative assessment (Figure 1). After a full-text review, 9 articles were excluded because the bond strength was not tested [46,47,48,49,50,51,52,53,54], leaving 78 publications for qualitative analysis. Of these, 10 studies were excluded from the quantitative analysis: 1 study did not present the data in the form of mean and SD [55], 3 studies did not have other studies for making comparisons [43,56,57], 3 studies did not contain a clearly defined control group [58,59,60], and in 3 articles, complete data could not be extracted [32,61,62]. Thus, 68 studies were included in the meta-analysis.

The qualitative synthesis of the studies included in this systematic review are summarized in Table S1. Both E&R and SE adhesives were identified; universal adhesives applied in both adhesive strategies were recognized too. Several strategies were identified, including the active application of the adhesive, multiple-layer application, the application of the adhesive using an electric current, an extended application time, the application of an extra hydrophobic resin layer, the application of the adhesive using an ultrasonic device, and a shortened application time. Most of the studies evaluated the bond strength after 24 h of aging, and for the long-term tests, both distilled water storage and thermocycling were identified. 

**Figure 2:**
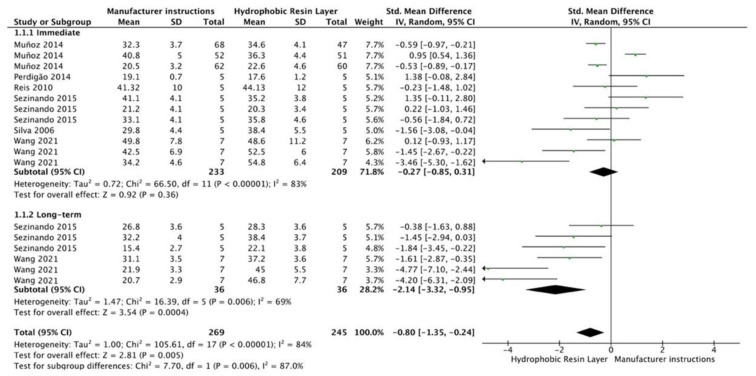
Forest plot showing the bond strength values when the adhesive was covered by a hydrophobic resin layer [63,64,65,66,67,68].

**Figure 3:**
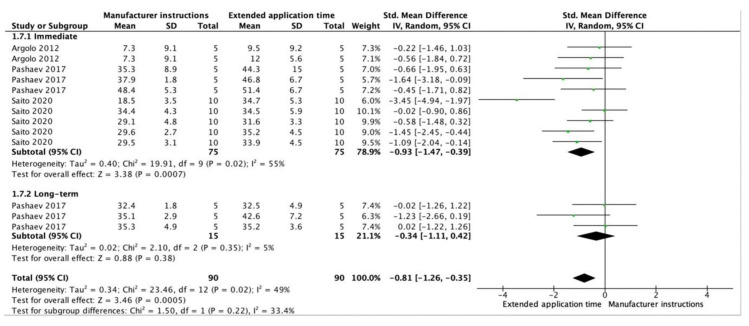
Forest plot showing the bond strength values when the adhesive was applied by extending their application time [69,70,71].

**Figure 4:**
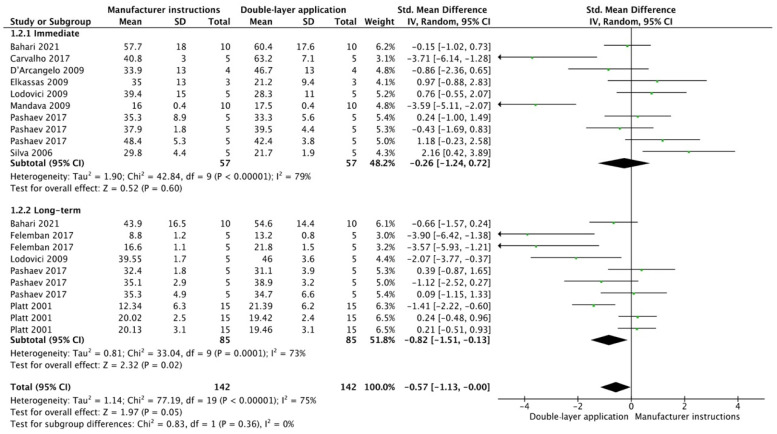
Forest plot showing the bond strength values when the adhesive was applied in two consecutive layers [63,69,72,73,74,75,76,77,78,79].

**Figure 5:**
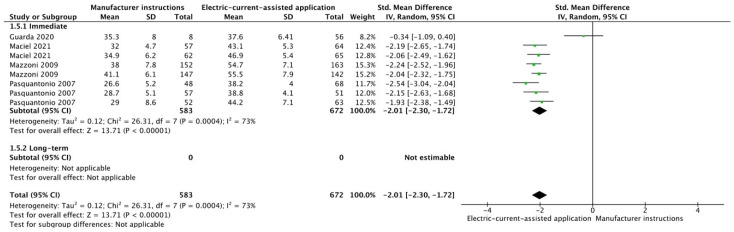
Forest plot showing the bond strength values when the adhesive was applied using an electric current [80,81,82,83].

**Figure 6:**
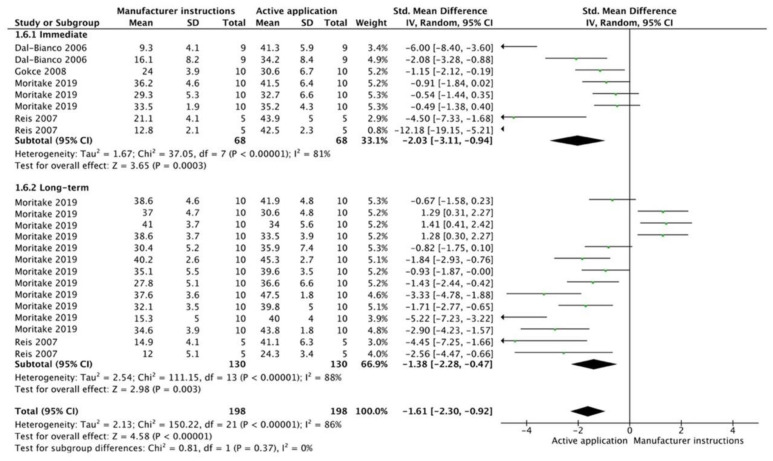
Forest plot showing the bond strength values when the adhesive was used in an active application [84,85,86,87].

**Figure 7:**
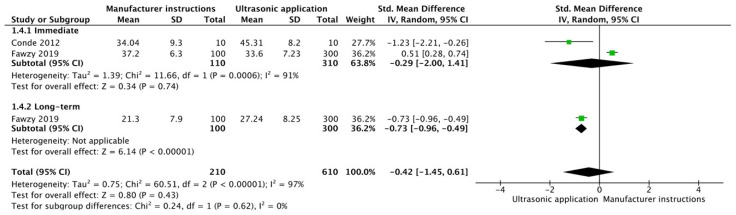
Forest plot showing the bond strength values when the adhesive was applied with an ultrasonic device [88,89].

**Figure 8:**
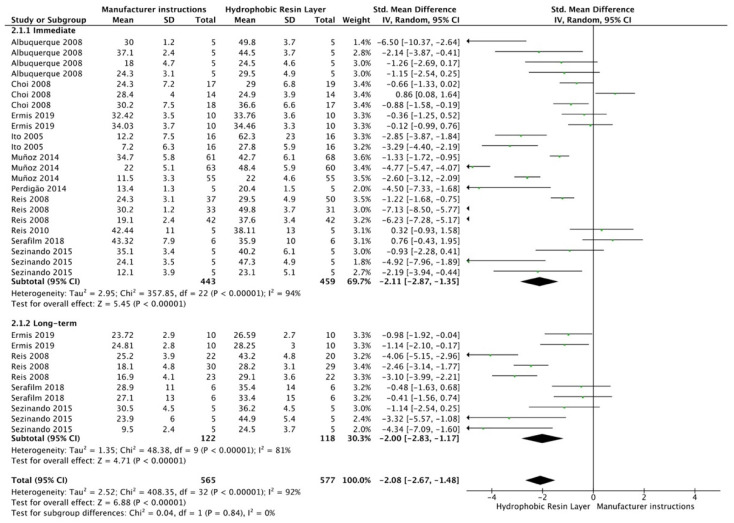
Forest plot showing the bond strength values when the adhesive was covered with a hydrophobic resin layer [64,65,66,90,91,92,93,94,95].

**Figure 9:**
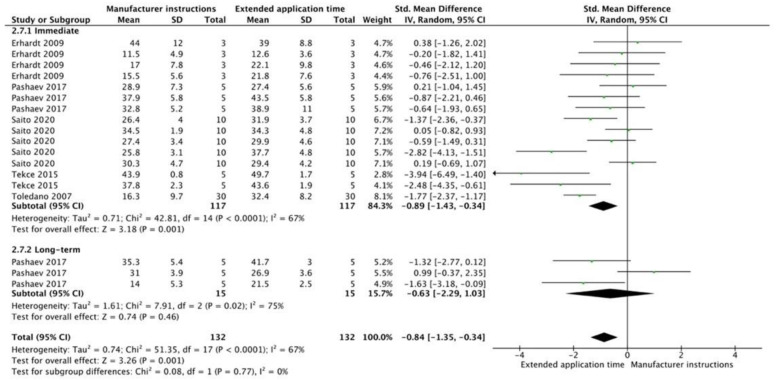
Forest plot showing the bond strength values when the adhesive was applied with an extended time application [69,71,96,97,98].

**Figure 10:**
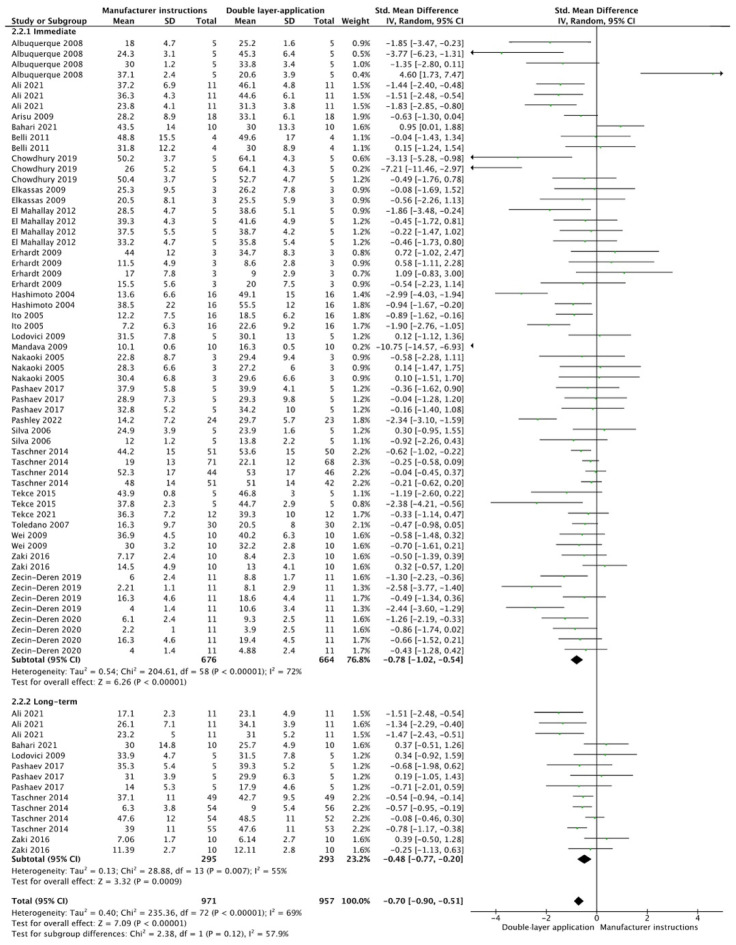
Forest plot showing the bond strength values when the adhesive was applied in two consecutive layers [63,69,72,75,77,78,92,93,96,97,98,99,100,101,102,103,104,105,106,107,108,109,110,111].

**Figure 11:**
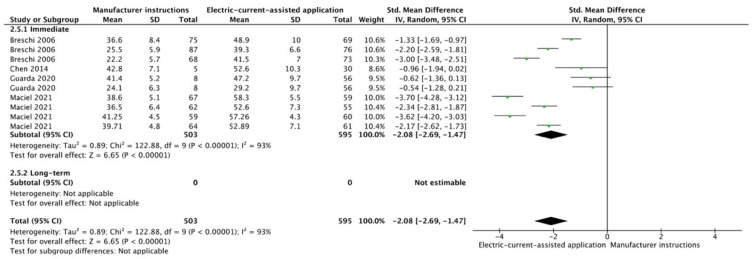
Forest plot showing the bond strength values when the adhesive was applied using an electric current [80,81,112,113].

**Figure 12:**
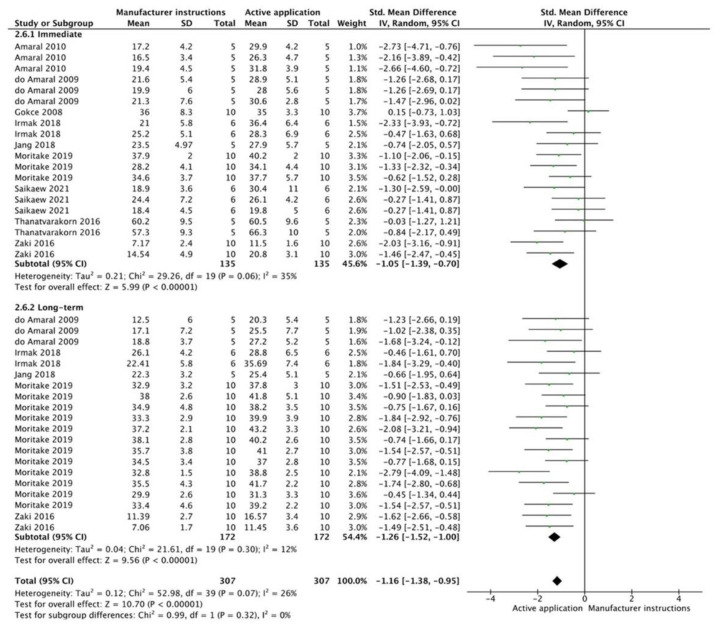
Forest plot showing the bond strength values when the adhesive was used in an active application [86,87,109,114,115,116,117,118,119].

**Figure 13:**
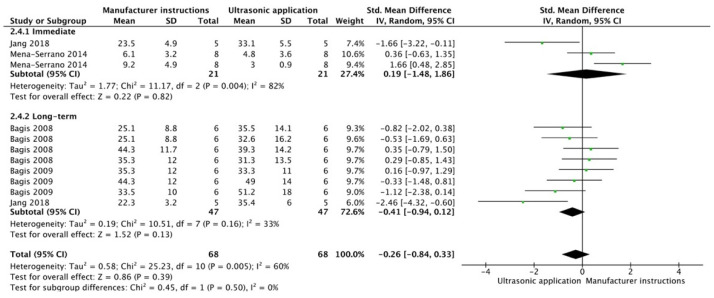
Forest plot showing the bond strength values when the adhesive was applied with an ultrasonic device [117,120,121,122].

**Figure 14:**
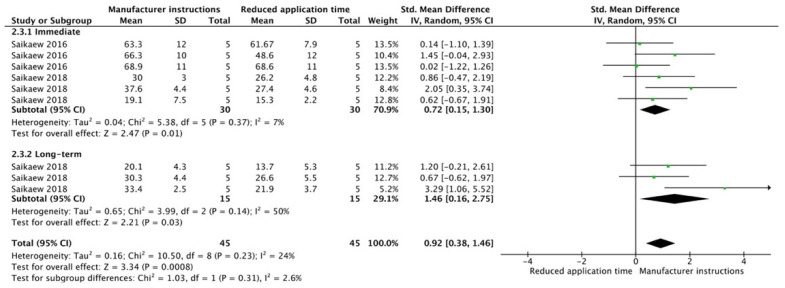
Forest plot showing the bond strength values when the adhesive was applied with reduced application time [123,124].

The results from the meta-analysis are shown in Figure 2, Figure 3, Figure 4, Figure 5, Figure 6, Figure 7, Figure 8, Figure 9, Figure 10, Figure 11, Figure 12, Figure 13 and Figure 14. Regarding E&R adhesive systems, the application modalities that improved the overall bond strength were the application of a hydrophobic resin layer (*p* = 0.005), an extended application time (*p* < 0.001), an application assisted by an electric current (*p* < 0.001), a double-layer application (*p* = 0.05), and the active application of the adhesive (*p* < 0.001). Worth mentioning is that the above-mentioned techniques improved, mostly, long-term bond strength. On the other hand, only the use of an ultrasonic device did not improve bond strength (*p* = 0.43) (Figure 2, Figure 3, Figure 4, Figure 5, Figure 6 and Figure 7).

For the SE adhesive systems, a similar behavior was observed. The techniques that improved the overall bond strength were the application of a hydrophobic resin layer (*p* < 0.001), an extended application time (*p* = 0.001), an application assisted by an electric current (*p* < 0.001), a double-layer application (*p* < 0.001), and the active application of the adhesive (*p* = 0.001). Once again, subgroup analysis showed that the statistically significant improvement was observed only for long-term bond strength. On the other hand, the use of an ultrasonic device did not show any improvement (*p* = 0.39); while the use of reduced application times impaired bond strength (*p* = 0.008) (Figure 8, Figure 9, Figure 10, Figure 11, Figure 12, Figure 13 and Figure 14).

According to the parameters for methodological quality assessment, most studies included were classified with medium risk of bias (Table S2). Most of the studies analyzed failed to report the single operator, operator blinded, and sample size calculation parameters.

## 4. Discussion

A systematic review and meta-analysis were directed concerning the assessment of the bond strength of adhesive systems to dentin depending on distinctive application modalities: an extra hydrophobic resin layer, an extended application time, a double-layer application, an application assisted by an electric current, the active application of the adhesive, the use of an ultrasonic device, and a reduced application time. Most of the application modalities improved dentin bond strength, while few impaired dentin bond strength. Considering this, the null hypothesis stating that the different application modalities would not provide similar bond strength to dentin when compared to the application of the adhesive according to the manufacturer’s instructions was partially rejected.

One of the utmost pertinent conducts to distinguish a commercial dentinal adhesive product is to measure the bond strength [125]. The most popular bond strength testing methods apply tensile or shear force; further, most adhesion strength examinations are executed on enamel or dentinal surfaces [126]. At the beginning of adhesive dentistry, shear and tensile bond strength tests were performed exclusively on specimens with relatively large, bonded areas [127]. Over the years, micro-bond strength tests have been developed claiming that smaller test specimens are “stronger” than larger ones due to the lower probability of the presence of critical sized defects [128]. Additionally, it was claimed that the long-term bond strength evaluation of dental adhesive systems would provide better clinical correlation since, under aging procedures, the resin–tooth interface could be prone to degradation, as occurs in a clinical scenario [1].

Appropriately, numerous methods have been argued to offer noteworthy benefits in enhancing dentinal bond strength, promoting resin infiltration, and solvent evaporation, and they were proposed depending on the application modalities [17]. The purpose of this study is to determine the influence of several changes to the manufacturer’s protocol application mode on dentinal bond strength.

The application of an extra bonding layer comprises an application of a supplementary layer of a hydrophobic resin to coat the adhesive [90]. The hydrophilic characteristic of the monomers inside some adhesives makes them permeable to water and compromises dentinal bonding durability. This is due to the affinity of the monomer for generating hydrogen bonds with the hydrophilic portion of acidic monomers existent in the mixture, which changes the formation of the polymer chains [91]. In an attempt to maintain the quality of the adhesive layer, many authors proved that the application of an extra bonding layer results in (1) higher bond strength, (2) higher hydrophobicity, (3) thicker adhesive layer, (4) better-sealed interface, (5) higher conversion rate, (6) better polymerization efficiency by a reduction in the detrimental effects of polymerization stress of composites resins, (7) better mechanical properties, and (8) less possible hydrolytic degradation of resin and collagen fibrils [49,129,130]. This strategy helps in stabilizing the adhesive interface against water sorption from the outer oral cavity and water ingress by osmosis from the dentin substrate [90,92]. SE adhesives are hydrophilic materials, so additional protection is necessary. Besides this, even E&R adhesives, especially those that are formulated using 2-hydroxyethyl methacrylate (HEMA), are also hydrophilic, so an extra hydrophobic layer can reduce the concentration of retained solvents and unreacted monomers [131]. Hence, to increase the bond strength of the adhesive–dentin interface [132], an extra layer of hydrophobic coat is preferred, as was demonstrated by this meta-analysis.

In the bonding approach to the dentin structure, it is deemed crucial to prolong the application time of adhesive systems for better bond performance and better monomer diffusion, and this is possible by enhancing the chemical interaction between monomers and HAp [93]. This approach increases the saturation of collagen fibrils by resin, as monomers preferably must plug the space between the exposed collagen [133,134]; otherwise, the performance and durability of adhesive systems might be compromised. Further, when a longer application time of the adhesive was achieved, more solvated monomers could evaporate, hereafter authorizing the advance of a stronger polymer within the dentinal surface and superior resin–dentin bond strength [69,135]. This statement is in agreement with this meta-analysis. Further, any recommendation for reducing the application times must be established on an extensive in vivo and in vitro documentation; therefore, it is powerfully logical to follow the manufacturers’ directions for adhesive application.

Another bonding application is the use of a multiple-layer application. A previous paper demonstrated that by using this approach, an increase in immediate dentin bond strength was achieved, but no improvement in bond strength was observed after aging [17]. The current trends in bonding appear to favor a single application of adhesive systems, but this could not create a thicker HL or adhesive layer in which micromechanical retention with the underlying composite resin exists. However, previous papers proposed that double or triple adhesive layers enhance dentin bond strength by improving monomer penetration into the HL and increasing chemical interactions [93,99]. Consequently, an additional layer application should be considered as a fundamental clinical step. Supplementarily, 10-MDP monomer needs an appropriate time of 20 s for its chemical interaction to take place; nevertheless, applying a second coat of such a monomer without curing the first one permits the first layer to adequately interact with HAp and, therefore, promotes additional bonding [136]. Further, it should be noted that the amplified dentin bond strength under double application is due to numerous mechanisms working concurrently. As the solvent inside adhesives is evaporated between each adhesive layer, the concentration of co-monomers that subsist after each layer application rises [93], consequently refining the quality of the HL [137]. A previous report [137] showed that there was an improvement in the dentin bond performance when multiple adhesive layers were applied but not cured. This cannot be accredited to the upsurge in adhesive layer thickness but nonetheless to the perfected quality of the adhesive layer. The thickness of the adhesive layer increases only when each coat of adhesive is light-cured. This safeguards that the demineralized dentinal substrate will be sufficiently protected, lessening the detrimental effects of oxygen inhibition by means of defective bond formation for both SE [93,100,138] and E&R adhesives [139]. All in all, double application layers are suggested for clinicians, and the material and substrate contents of each adhesive should be taken into consideration.

Additionally, an application assisted by an electric current and the active application of adhesive systems enhanced the dentin bond performance of the etching mode by facilitating the penetration of adhesives into the branches of dentinal tubules. Further, an adhesive application protocol based on the use of an electric signal has been introduced by some investigators [29,52,80,81,112]. This technique improves the chemical interaction between adhesive systems and tooth structures and, thus, increases monomer infiltration of the demineralized dentinal substrate by altering the surface charges and hydrogen bonding potential of the dentin substrate [82,112,113,140]. Consequently, there is an enhancement of dentinal wettability, leading to solvent evaporation [112]. Furthermore, electrically assisted methods enhance dentin bond strength and reduce nanoleakage in the HL [82,83,112,140]. However, it requires a special device for the adhesive application that releases adhesive by an electrical potential difference between the adhesive system and tooth surfaces [113]. Resin monomer diffusion into etched dentin could be enhanced through the use of electric current, as polar constituents, such as HEMA, polyalkanoic acid, biphenyl dimethacrylates copolymers, and dipentaerythritol penta-acrylate phosphate, contained in the adhesive formulation might interact with the electric field [82]. It has been formerly stated that electrical currents endorse the movement of an ionized substrate [141,142]. Though, it is still uncertain whether suitable dentin hybridization could be formed by easing the impregnation of ionized substrates in diverse conditions and enhancing the removal of water for SE adhesives [143,144]. Next, the application of an electric current might also increase the water substitution rate by means of the modification of water dipoles, thus, preferring water–solvent exchange throughout resin infiltration [90]. It has been claimed that the application of electric currents between 30 and 35 μA meaningfully improved bond strength and bonding quality [80]. This could be considered safe, as bonding procedures performed with 35 μA could not affect cell viability [113]. Ideally, as suggested by this analysis, the use of an electric current during the application of E&R and SE adhesive systems has been claimed to increase the bonding of an adhesive to dentin by enhancing substrate impregnation.

Agitation can deliver a reliable etch; furthermore, it can enhance the interaction between the acidic monomers and dental substrate [145]. Actually, the active application of adhesives using a scrubbing technique leads to the impregnation of a higher rate of monomers inside the smear layer and facilitates solvent evaporation, hence, improving adhesive interface quality. This technique does not require any supplementary step [146]. In addition, agitation preserves freshly acidic solution in interactions with the tooth substrate. Hence, improving the immediate bond performance of the simplified E&R adhesive systems [37,84]. It seems that previous reports suggested that higher bond strength was achieved when a two-step SE adhesive system was used. This could be explained by the complete dissolution of the smear layer into the adhesive [147]. On the other hand, a dissimilar finding was detected by Miyazaki et al. when testing the same adhesion strategy [145]. Another study proposed that the performance of SE adhesive systems was related to the agitation duration time [60]. All in all, adhesive agitation was able to increase the moieties’ kinetics and permit better monomer diffusion inward; however, the solvents spread outward [85].

On the other hand, it seems that the use of an ultrasonic device did not show any improvement for both SE and E&R adhesive systems in this analysis. The ultrasonic mode might not enhance the dentinal bond performance outcomes of some SE adhesive systems. Ultrasonics is a division of acoustics concerned with the help of sound vibrations that possess a frequency range above the audible level. One should state that the frequency in the oscillating instruments in dental practice is ultrasonic for frequencies ranging from 20,000 to 40,000 Hz. In addition, it is considered sonic when the frequency ranges from 1000 to 6000 Hz [120]. The application of ultrasonic agitation energy could induce acoustic streaming and upsurge infiltration of the dentin adhesive into the dentinal substrate. This conclusion seems to not support the results of this meta-analysis, as improvement in dentin bond strength was not observed in all adhesives tested.

The analysis of this review exhibited that the use of reduced application times impaired the dentin bond strength of SE adhesive systems. Currently, clinicians begin to escalate the settlements related to step reduction in both E&R [148] and SE adhesives [149]. A recently launched adhesive system by manufacturers, named G-Premio Bond universal adhesive (GC Corporation, Tokyo, Japan), delivers dental clinicians an alternative to the SE approach for adhesion to dentin without the need to wait for the adhesive to interact with the bonding substrate (no-waiting SE; Japanese version of manufacturer’s instructions), or the interaction occurs after leaving the adhesive undisturbed for 10 s (10 s SE; international version of manufacturer’s instructions) [123]. This new concept of a no-waiting adhesive, while pleasing to many clinicians, must not compromise the performance and durability of adhesion to dentin. The author concluded that the no-waiting SE approach prior to adhesive polymerization might gave sufficient bond strength. Nevertheless, extended application times with the 10 s SE mode instead of no-waiting mode advances its short-term dentinal bonding performance [56]. Hence, applying a SE adhesive for shorter times than recommended by the manufacturer might not represent the best use of the adhesive. Dentists normally work without using a stopwatch to precisely time the individual application steps, and they demand a decrease in the uncomfortable treatment time for their patients. Consequently, it is expected that clinicians do not always follow the application times specified by the instructions of the manufacturers [150], and these deviations from the suggested application times could hinder the dentin bond strength of adhesive systems [151], accordingly compromising the longevity of dental resin restorations. As was proved by Hardan et al. [17], a reduced application time weakens both the immediate and aged bond strength of universal adhesives applied to dentin with E&R or SE modes. As a consequence, this modification should not be followed by clinicians.

As materials in close contact with biological tissues, both directly and indirectly, bio- compatibility is one of the utmost serious desires for adhesive systems [152]. It has been established that the constituents of dental adhesives, such as TEGDMA (triethylene glycol dimethacrylate) and HEMA, might diffuse throughout the dentinal tubules and extend into the pulp tissue in concentrations considered toxic to pulp cells [153]. It is worth mentioning that the increase in bond strength observed for some of the described techniques in this review is based on the increase in the degree of conversion of the materials [129]. This increase in the degree of conversion could be related to a reduction in the release of residual monomers, leading to an improvement in the biocompatibility of dental adhesives [19], and this relationship should be addressed in future works.

The present systematic review and meta-analysis distinguished the effect of different application modalities on the bonding ability of adhesive systems to dentin when compared to the application of the adhesive according to the manufacturer’s instructions. The outcomes of this review should be considered with caution as, in clinical conditions, a wet environment could lead to rapid adhesive–dentinal interface degradation. Therefore, future studies should be performed to evaluate the solubility, degree of conversion, shelf-life stability, and polymerization rate of the adhesive applied with different application modalities. Moreover, additional studies on cytotoxicity are recommended in order to analyze the best intensity when using an electric signal for adhesive application. Future reports must be directed, particularly randomized controlled clinical trials, with the drive to acquire the performance of different adhesive systems in the clinical performance of resin-based restorations to the dentinal structure. Additionally, little information exists concerning the influence of these strategies on enamel bond strength. Thus, testing these application modalities on enamel substrates could be important in future investigations. The relationship between the chemical composition of adhesive systems and these modalities are of extreme importance. Therefore, research should be directed towards testing more adhesive systems and more strategies in an attempt to achieve better bonds on tooth substrates. In this review, the best scientific evidence available regarding the dentin bonding efficacy of adhesive systems applied using different application modalities was compiled. To date, the monotonous use of simplified adhesive systems in combination with composite resin to restore dentinal margins is still doubtful and challenging. Consequently, it seems that establishing a durable and stable dentin bond by means of different application modalities could be possible when properly used. Hence, these approaches are crucial for the long-term clinical achievement of restorative treatment to dentin. All in all, essential steps also exist in the application process of various adhesive agents that necessitate recognizing the chemistry of the adhesive being used. Existing application modalities were presented in this study to help clinicians preserve the durability of the bond to the dentin substrate. Nonetheless, no agent or material can overcome poor procedures.

## 5. Conclusions

Within the limitations of this systematic review of in vitro studies, the findings suggest that the application of adhesive systems using application modalities that are different than the manufacturers’ recommendations may increase the bond strength of resin-based materials to dentin. The application modalities that favored the overall bond strength to dentin were an extended application time, a double-layer application, an application assisted by an electric current, the active application of the adhesive, and the application of a hydrophobic resin layer.

## Figures and Tables

**Table 1:** Search strategy used in PubMed.

#1 Dentin OR Dentine
#2 Bonding OR Bond OR Bonding efficacy OR Dental bonding OR bond strength OR bonding effectiveness OR Bonding performance OR Bond performance OR adhesive #2 properties OR Micro-tensile strength OR microtensile strength OR Microtensile bond strength OR bonding properties OR microshear bond strength OR shear bond strength OR performance
#3 double-application coat OR double-application time OR Bonding application time OR Time factors, working time OR adhesive dental coating OR rubbing OR agitation OR scrubbing OR application mode OR double application OR single application OR Double-layer application OR adhesive layer OR ultrasonic OR agitation OR vibration OR ultrasonics OR application time OR Double application technique OR adhesive dental coating
#1 AND #2 AND #3

## Supplementary Materials

The following supporting information can be downloaded at: https://www.mdpi.com/article/10.3390/cells12010190/s1, Table S1: Demographic data of the included studies; Table S2: Risk of bias assessment. References [63,64,65,66,67,70,71,72,73,74,75,76,77,78,79,86,87,88,89,94,95,96,97,98,101,102,103,104,105,106,107,108,109,110,111,114,115,116,117,118,119,121,122,124,154,155,156,157] are cited in Supplementary Materials.
