# Supplementary material for: Effect of Different Application Modalities on the Bonding Performance of Adhesive Systems to Dentin: A Systematic Review and Meta-Analysis
Source: Cells. 2023 Jan 3;12(1):190. doi: 10.3390/cells12010190 (PMC9818277; doi:10.3390/cells12010190)
Supplement: Supplementary file 1 [file cells-12-00190-s001.zip › cells-2065390-supp.pdf]

cells MDPI

**Table S1.** Demographic data of the included studies.

| Study and year | Application modality | Outcomes evaluated | Test used | Storage conditions |
|---|---|---|---|---|
| Albuquerque, 2008 [91] | Double application<br>Hydrophobic Layer | Bond strength<br>Failure mode | µTBS | Distilled water (24 h/37°C) |
| Amaral, 2010 [113] | Agitation on dentin<br>Passive application | Bond strength<br>Failure mode<br>Silver Nitrate Uptake | µTBS | Distilled water (24 h/37°C) |
| Argolo, 2012 [69] | Prolonged photoactivation time | Bond strength<br>Degree of conversion | µSBS | Distilled water (24 h/37°C) |
| Asmussen, 2003 [153] | Application of adhesive with or without light curing | Bond strength | SBS | 1 day to 1 year in water (37°C) |
| Amsler, 2015 [55] | Reduced application time | Bond strength<br>Failure mode | SBS | Black photoresistant boxes in an incubator (UM 500, Memmert; Schwabach, Germany) at 37°C and 100% humidity for 24 h. |
| Arisu, 2009[100] | Multiple consecutive applications | Bond strength<br>Failure mode | µTBS | Distilled water (24 h/37°C) |
| Ali, 2021[101] | Double layer application | Bond strength<br>Failure mode | µTBS | Sterile water (24 h/37°C)<br>Sterile water (12 months/37°C) |
| Bagis, 2009 [120] | High Frequency Ultrasonic Agitation | Bond strength<br>Failure mode<br>Scanning electron micrographs (SEM) | µTBS | Distilled water (72 h/37°C) |
| Bagis, 2008 [121] | Ultrasonic Agitation | Bond strength<br>Failure mode | µTBS | Distilled water (72 h/37°C) |
| Bahari, 2021 [71] | Double application | Bond strength | SBS | Distilled water (24 h/37°C)<br>Thermal cycling was performed by applying 3000 cycles at 5°C–55°C<br>10% sodium hypochlorite for 3 h. |

| | | | | |
|---|---|---|---|---|
| Belli, 2011 [102] | Multiple Coats | Bond strength<br>Failure mode<br>SEM | µTBS | Distilled water (24 h/37°C) |
| Breschi,2006 [111] | Electric-current-assisted Application | Bond strength<br>Failure mode<br>Nanoleakage evaluation | µTBS | De-ionized water for 24 h. |
| Burrer, 2020 [57] | Application time | Bond strength<br>Failure mode | µTBS | Not mentioned |
| Cardoso, 2005 [58] | Prolonged application time | Loss of Mass<br>Bond strength<br>Failure mode | µTBS | Distilled water (24 h/37°C) |
| Carvalho, 2017 [72] | Multiple adhesive layering | Bond strength<br>Failure mode<br>Silver nitrate uptake | µTBS | 24 h storage |
| Chasqueira, 2013 [32] | Additional adhesive layers<br>Extra hydrophobic adhesive layer | Bond strength | SBS | 24 h/37°C and 100% humidity |
| Chasqueira, 2020 [62] | Extra layers<br>Hydrophoibic resin layer | Bond strength<br>Failure mode<br>Nanoleakage | SBS | Water (24 h/37°C)<br>Sodium azide solution for a period of 6 months or 18 months |
| Chen, 2014 [112] | Direct current intensities | Bond strength<br>Failure mode<br>Nanoleakage evaluation<br>Cell isolation and culture<br>Fluorescence microscopy<br>Cell proliferation | µTBS | Distilled water (24 h/37°C) |
| Choi, 2008 [93] | Additional coat of intermediate hydrophobic resin | Bond strength<br>Nanoleakage | µSBS | 24 h storage |
| Chowdhury, 2019 [98] | Double application | Bond strength<br>Failure mode<br>Hardness | µTBS | Distilled water (24 h/37°C) |
| Conde, 2012 [87] | Two layers | Bond strength<br>Failure mode<br>Hybrid layer analysis<br>Fractographic analysis | µTBS | Distilled water (24 h/37°C) |
| D'Arcangelo, 2009 [73] | Multiple layers | Adhesive layer thickness measurement<br>Bond strength | µTBS | Distilled water (24 h/37°C) |

| | | | | |
|---|---|---|---|---|
| | | Failure mode | | |
| Dal-Bianco, 2006 [84] | Rubbing action | Bond strength<br>Failure mode | µTBS | Distilled water (24 h/37°C) |
| Silva, 2006 [63] | Additional adhesive application | Bond strength<br>Failure mode<br>Adhesive layer thickness measurements | µTBS | Distilled water (24 h/37°C) |
| do Amaral, 2009 [114] | Active application | Bond strength<br>Failure mode<br>Nanoleakage evaluation | µTBS | Distilled water (24 h/37°C)<br>Distilled water (6 months /37°C) |
| El Mahallay, 2012 [103] | Double application | Bond strength<br>Failure mode | µTBS | Distilled water (24 h/37°C) |
| Erhardt, 2009 [95] | Double layering and prolonged application time | Bond strength<br>Failure mode<br>SEM | µTBS | Distilled water (24 h/37°C) |
| Elkassas, 2009 [74] | Number of applications | Bond strength<br>Failure mode<br>SEM | µTBS | Deionized water at 37°C for one week |
| Ermis, 2019 [89] | Extra hydrophobic adhesive layer | Bond strength<br>Failure mode<br>SEM | µTBS | Distilled water (24 h/37°C)<br>Distilled water (6 months /37°C) |
| Felemban, 2017 [75] | Adhesive layers | Bond strength | µSBS | Water (24 h/37°C) and thermocycling 500x in a 5° – 55°C water bath with a dwell time of 30 s |
| Fawzy, 2019 [88] | High intensity focused ultrasound | Atomic Force Microscopy<br>SEM/TEM investigation<br>Proteases detection<br>Raman analysis<br>Resin-Dentin bonding<br>Microscopic investigation of resin-dentin interface<br>Bond-strength testing<br>Failure mode | µTBS | 24 h or 12 months in artificial-saliva at 37 ◦ C |
| Gokce, 2008 [88] | Application of adhesive with agitation | Bond strength<br>SEM | SBS | Distilled water (24 h/37°C) |

| | | | | |
|---|---|---|---|---|
| Guarda, 2020 [79] | Electric current-assisted application | Bond strength<br>Failure mode<br>Confocal laser scanning microscopy (CLSM) | µTBS | Distilled water (24 h/37°C) |
| Hardan, 2022 [59] | Active bonding application (short and long-time application) | Bond strength<br>Failure mode<br>SEM | µTBS | Distilled water (24 h/37°C)<br>Distilled water (6 months /37°C) |
| Hashimoto, 2004 [104] | Multiple adhesive coatings | Bond strength<br>Nanoleakage | µTBS | Distilled water (24 h/37°C) |
| Huang, 2017 [56] | No-waiting self-etch | Bond strength<br>Failure mode<br>Transmission electron microscopy (TEM)<br>Attenuated total reflection-Fourier transform infrared spectroscopy | µTBS | Deionized water at 37 °C for 24 h<br><br>Thermomechanical challenge, using 10,000 thermal cycles and 240,000 mechanical cycles |
| Irmak, 2018 [115] | Rubbing force magnitude | Bond strength<br>Failure mode | µTBS | Thermal aging in distilled water for 10,000 cycles |
| Ito, 2005 [92] | Multiple Coatings | Bond strength<br>TEM of Nanoleakage | µTBS | Water (24 h/37°C) |
| Jang, 2018 [116] | Various agitation methods | Bond strength<br>Fracture mode<br>TEM | µTBS | Distilled water (24 h/37°C)<br>Aging treatment |
| Karalar, 2021 [154] | Adhesive application time | Bond strength<br>Failure mode<br>SEM | SBS | Distilled water (24 h) |
| Kerekes-mathe, 2020 [61] | Adhesive application methods (two layers) | Bond strength | µSBS | Not mentionned |
| King, 2005 [155] | Multiple coats | TEM<br>Fluid conductance of dentin<br>SEM<br>Bond strength | µTBS | Distilled water (24 h/37°C) |
| Lodovici, 2009 [76] | Adhesive thickness (Two layers) | Bond strength<br>Fracture mode<br>SEM | µTBS | Thermal/mechanical cycling |
| Maciel, 2021 [80] | Electric current application | Bond strength<br>Fracture pattern<br>Confocal laser scanning microscopy interfacial characterization | µTBS | Distilled water (24 h/37°C) |

| | | | | |
|---|---|---|---|---|
| Mandava, 2009 [77] | Multiple consecutive applications | Bond strength | TBS | Not mentionned |
| Mazzoni, 2009 [82] | ElectroBond-assisted application | Bond strength<br>Failure mode<br>Nanoleakage evaluation | µTBS | Deionized water for 24 h |
| Mena-Serrano, 2014 [119] | Sonic application mode | Measurement of hydraulic conductance of dentin<br>Bond strength<br>Failure mode | µTBS | Distilled water (24 h/37°C) |
| Moritake, 2018 [86] | Active application | Thermal Cycling (TC)<br>Bond strength<br>Failure mode<br>SEM | SBS | Distilled water (24 h/37°C), then 5000, 10,000, 30,000, or 50,000 cycles of thermocycling |
| Muñoz, 2014 [64] | Hydrophobic resin coating | Bond strength<br>Failure mode<br>Nanoleakage<br>Degree of conversion in situ | µTBS | Distilled water (24 h/37°C) |
| Nakaoki, 2005 [105] | Double-application | Bond strength<br>Fracture mode<br>SEM | µSBS | Water (24 h/37°C) |
| Pashaev, 2017 [68] | Double-coating and times | Bond strength<br>Fracture mode<br>SEM | µTBS | Distilled water (24 h/37°C)<br>Distilled water (6 months /37°C) |
| Pashley, 2002 [142] | Two applications | Bond strength<br>Failure mode<br>Ultrastructural examination of resin–dentine interfaces<br>Silver-staining technique | µTBS | Distilled water (24 h/37°C) |
| Pasquantonio, 2007 [81] | Electric device | Bond strength<br>Failure mode<br>FE-SEM nanoleakage evaluation | µTBS | Deionized water for 24h |
| Perdigão, 2014 [66] | Hydrophobic resin coat | Bond strength<br>Failure mode<br>Degree of conversion in situ | µTBS | Distilled water (24 h/37°C) |
| Platt, 2001 [78] | Double adhesive application | Bond strength<br>Fracture mode<br>Confocal microscopy | SBS | Distilled water at 36°C and thermocycling. |
| Reis, 2008 [94] | Extra layer of hydrophobic resin | Bond strength<br>Failure mode<br>SEM | µTBS | Distilled water (24 h/37°C) |

| | | | | |
|---|---|---|---|---|
| | | | | Distilled water (6 months /37°C) |
| Reis, 2008 (b) [134] | Prolonged application times | Bond strength<br>Failure mode | µTBS | Distilled water (24 h/37°C)<br>3 years of water storage |
| Reis, 2007 [83] | Rubbing of adhesive | Bond strength<br>Fracture mode | µTBS | Distilled water (24 h/37°C)<br>1 year of water storage |
| Reis, 2010 [65] | Extra thin layer | Bond strength<br>Failure mode | µTBS | Distilled water (24 h/37°C) |
| Saikaew, 2016 [122] | Reduced application time | Bond strength<br>Fracture mode<br>Interfacial structure observation | µTBS | Water (24 h/37°C) |
| Saikaew, 2021 [117] | Active application mode | Bond strength<br>Failure mode<br>Adhesive-dentin interface observation | µTBS | Distilled water (24 h/37°C) |
| Saikaew, 2018 [123] | Shortened application time | Bond strength<br>Fracture mode<br>TEM observation of resin-dentin interface | µTBS | Water (24 h/37°C)<br>Distilled water (1 year/37°C) |
| Saito, 2019 [70] | Prolonged application time | Bond strength<br>Failure mode<br>Surface free energy | SBS | Distilled water (24 h/37°C) |
| Scheffel, 2014 [43] | Time between adhesive application and photoactivation | Bond strength<br>Light trichrome staining<br>Analysis by light microscopy | µTBS | Deionized water (24 h/37°C) |
| Serafim, 2018 [90] | Hydrophobic layer | Bond strength<br>Fracture mode | µTBS | Distilled water (24 h/37°C)<br>Distilled water (3 months/37°C)<br>Distilled water (6 months/37°C) |
| Sezinando, 2015 [66] | Hydrophobic resin coating | Bond strength<br>Failure mode<br>Nanoleakage | µTBS | Distilled water (24 h/37°C)<br>Distilled water (6 months/37°C) |
| Silva, 2006 [63] | Additional adhesive application | Bond strength<br>Failure mode<br>Adhesive layer thickness measurements | µTBS | Distilled water (24 h/37°C) |

| | | | | |
|---|---|---|---|---|
| Taschner, 2014 [106] | Double-layer application | Bond strength<br>Failure mode<br>SEM | μTBS | Artificial saliva (24 h/37°C)<br>Artificial saliva (6 months/37°C)<br>Five hours in 10 % sodium hypochlorite at room temperature |
| Tekce, 2021 [156] | Double-layer application | Bond strength<br>Failure mode<br>SEM | SBS | Distilled water (24 h/37°C) |
| Tekçe, 2015 [96] | Doubling adhesive application time<br>Doubling adhesive coating | Bond strength<br>Failure mode<br>SEM | μTBS | Distilled water (24 h/37°C) |
| Thanatvarakorn, 2016 [118] | Scrubbing technique | Bond strength<br>Failure mode<br>SEM<br>Nanoleakage evaluation by TEM | μTBS | Water (24 h/37°C) |
| Toledano, 2007 [97] | Doubling application time<br>Doubling the number of adhesive coats | Bond strength<br>Failure mode | μTBS | Distilled water (24 h/37°C) |
| Velasquez, 2006 [60] | Placement agitation and placement time | Bond strength | SBS | Distilled water at room temperature |
| Wang, 2020 [67] | Hydrophobic coating | Bond strength<br>Fracture mode<br>SEM | μTBS | Distilled water (24 h/37°C) |
| Wei, 2009 [107] | Double-application | Bond strength<br>Failure mode<br>Naonindentation tests<br>SEM | μSBS | Water (24 h/37°C) |
| Zaki, 2016 [108] | Active application<br>Double adhesive layer | Bond strength<br>Failure mode | μSBS | Artificial saliva (24 h)<br>Artificial saliva (3 months) |
| Zecin-Deren, 2020 [109] | Multiplying applications and extending application time | Bond strength<br>SEM and energy dispersive spectrometry | SBS | Saline (24h) |
| Zecin-Deren, 2019 [110] | Multi-layer application | Bond strength<br>SEM and energy dispersive spectrometry | SBS | Saline (24h) |

Microtensile bond strength (μTBS); Micro-shear bond strength (μSBS); Shear bond strength (SBS).

**Table S2.** Risk of bias assessment.

| Study and year | Specimen randomization | Single operator | Operator blinded | Control group | Standardized specimens | Failure mode | Manufacturers' instructions | Sample size calculation | Risk of bias |
|---|---|---|---|---|---|---|---|---|---|
| Albuquerque, 2008 [91] | YES | YES | NO | YES | YES | YES | YES | NO | Medium |
| Amaral, 2010 [113] | NO | YES | NO | YES | YES | YES | YES | NO | Medium |
| Argolo, 2012 [69] | YES | NO | NO | YES | YES | NO | YES | NO | Medium |
| Asmussen, 2003 [153] | NO | NO | NO | YES | NO | NO | YES | NO | High |
| Amsler, 2015 [55] | NO | NO | NO | YES | YES | YES | YES | YES | Medium |
| Arisu, 2009[100] | NO | NO | NO | YES | NO | YES | YES | NO | High |
| Ali, 2021[101] | NO | NO | NO | YES | NO | YES | YES | YES | Medium |
| Bagis, 2009 [120] | YES | NO | NO | YES | YES | YES | YES | NO | Medium |
| Bagis, 2008 [121] | YES | NO | NO | YES | YES | YES | YES | NO | Medium |
| Bahari, 2021 [71] | YES | NO | NO | YES | YES | NO | YES | YES | Medium |
| Belli, 2011 [102] | YES | NO | NO | YES | NO | YES | YES | NO | Medium |
| Breschi,2006 [111] | NO | YES | YES | YES | YES | YES | YES | NO | Medium |
| Burrer, 2020 [57] | YES | NO | NO | YES | YES | YES | YES | NO | Medium |
| Cardoso, 2005 [58] | NO | NO | NO | NO | YES | YES | NO | NO | High |
| Carvalho, 2017 [72] | YES | YES | NO | YES | NO | YES | YES | NO | Medium |
| Chasqueira, 2013 [32] | YES | NO | NO | YES | YES | NO | YES | NO | Medium |
| Chasqueira, 2020 [62] | YES | NO | NO | YES | YES | YES | YES | YES | Medium |
| Chen, 2014 [112] | YES | NO | NO | YES | YES | YES | YES | NO | Medium |
| Choi, 2008 [93] | NO | NO | NO | YES | NO | NO | YES | NO | High |
| Chowdhury, 2019 [98] | YES | NO | NO | YES | YES | YES | YES | NO | Medium |
| Conde, 2012 [87] | YES | NO | NO | YES | NO | YES | NO | NO | High |
| D'Arcangelo, 2009 [73] | YES | NO | NO | YES | YES | YES | YES | NO | Medium |
| Dal-Bianco, 2006 [84] | YES | YES | NO | YES | NO | YES | YES | NO | Medium |
| Silva, 2006 [63] | YES | NO | NO | YES | NO | YES | YES | NO | Medium |
| do Amaral, 2009 [114] | YES | YES | NO | YES | YES | YES | YES | NO | Medium |
| El Mahallay, 2012 [103] | YES | NO | NO | YES | YES | YES | YES | NO | Medium |
| Erhardt, 2009 [95] | YES | NO | NO | YES | NO | YES | YES | NO | Medium |
| Elkassas, 2009 [74] | NO | NO | NO | YES | NO | YES | YES | NO | High |
| Ermis, 2019 [89] | YES | NO | NO | YES | YES | YES | YES | NO | Medium |
| Felemban, 2017 [75] | NO | NO | NO | YES | YES | NO | YES | NO | High |
| Fawzy, 2019 [88] | YES | NO | NO | YES | YES | YES | YES | NO | Medium |
| Gokce, 2008 [88] | YES | YES | YES | YES | YES | NO | YES | NO | Medium |
| Guarda, 2020 [79] | YES | NO | NO | YES | NO | NO | YES | NO | High |
| Hardan, 2022 [59] | YES | NO | NO | YES | YES | YES | YES | NO | Medium |
| Hashimoto, 2004 [104] | YES | NO | NO | YES | YES | YES | YES | NO | Medium |
| Huang, 2017 [56] | NO | NO | NO | YES | NO | NO | YES | NO | High |
| Irmak, 2018 [115] | YES | NO | NO | YES | NO | YES | YES | NO | Medium |
| Ito, 2005 [92] | YES | YES | NO | YES | YES | YES | YES | NO | Medium |
| Jang, 2018 [116] | NO | NO | NO | YES | NO | NO | YES | NO | High |
| Karalar, 2021 [154] | YES | NO | NO | YES | YES | YES | YES | NO | Medium |
| Kerekes-mathe, 2020 [61] | YES | NO | NO | YES | YES | YES | YES | NO | Medium |
| King, 2005 [155] | NO | NO | NO | YES | YES | NO | YES | NO | High |
| Lodovici, 2009 [76] | NO | NO | NO | YES | NO | NO | YES | NO | High |
| Maciel, 2021 [80] | NO | YES | NO | YES | YES | YES | YES | NO | Medium |
| Mandava, 2009 [77] | YES | NO | NO | YES | YES | YES | YES | YES | Medium |
| Mazzoni, 2009 [82] | YES | NO | NO | YES | NO | NO | YES | NO | High |

| | | | | | | | | | |
|---|---|---|---|---|---|---|---|---|---|
| Mena-Serrano, 2014 [119] | YES | NO | YES | YES | YES | YES | YES | NO | Medium |
| Moritake, 2018 [86] | YES | NO | NO | YES | YES | YES | YES | NO | Medium |
| Muñoz, 2014 [64] | NO | NO | NO | YES | NO | YES | YES | YES | Medium |
| Nakaoki, 2005 [105] | YES | YES | YES | YES | YES | YES | YES | NO | Low |
| Pashaev, 2017 [68] | YES | NO | NO | YES | NO | YES | YES | NO | Medium |
| Pashley, 2002 [142] | YES | YES | NO | YES | YES | YES | YES | NO | Medium |
| Pasquantonio, 2007 [81] | YES | NO | NO | YES | NO | YES | YES | NO | Medium |
| Perdigão, 2014 [66] | YES | YES | YES | YES | YES | YES | YES | NO | Low |
| Platt, 2001 [78] | YES | NO | NO | YES | YES | YES | YES | NO | Medium |
| Reis, 2008 [94] | NO | NO | NO | YES | YES | YES | YES | NO | Medium |
| Reis, 2008 (b) [134] | NO | YES | NO | YES | YES | YES | YES | NO | Medium |
| Reis, 2007 [83] | NO | NO | NO | NO | YES | YES | NO | NO | High |
| Reis, 2010 [65] | YES | YES | NO | YES | YES | YES | YES | NO | Medium |
| Saikaew, 2016 [122] | YES | YES | NO | YES | YES | YES | YES | YES | Low |
| Saikaew, 2021 [117] | YES | NO | NO | YES | NO | YES | YES | NO | Medium |
| Saikaew, 2018 [123] | YES | NO | NO | YES | YES | YES | YES | NO | Medium |
| Saito, 2019 [70] | YES | NO | NO | YES | NO | YES | YES | NO | Medium |
| Scheffel, 2014 [43] | NO | NO | NO | YES | YES | YES | YES | YES | Medium |
| Serafim, 2018 [90] | YES | NO | NO | YES | NO | NO | YES | NO | High |
| Sezinando, 2015 [66] | YES | NO | NO | YES | YES | YES | YES | NO | Medium |
| Silva, 2006 [63] | YES | NO | YES | YES | YES | YES | YES | NO | Medium |
| Taschner, 2014 [106] | YES | NO | NO | YES | NO | YES | YES | NO | Medium |
| Tekce, 2021 [156] | NO | NO | NO | YES | YES | YES | YES | NO | Medium |
| Tekçe, 2015 [96] | YES | NO | NO | YES | YES | YES | YES | NO | Medium |
| Thanatvarakorn, 2016 [118] | YES | NO | NO | YES | NO | YES | YES | NO | Medium |
| Toledano, 2007 [97] | NO | NO | NO | YES | YES | YES | YES | NO | Medium |
| Velasquez, 2006 [60] | NO | NO | NO | YES | NO | YES | YES | NO | High |
| Wang, 2020 [67] | NO | NO | NO | YES | NO | NO | YES | NO | High |
| Wei, 2009 [107] | YES | NO | NO | YES | YES | YES | YES | NO | Medium |
| Zaki, 2016 [108] | YES | NO | NO | YES | YES | YES | YES | NO | Medium |
| Zecin-Deren, 2020 [109] | YES | NO | NO | YES | YES | YES | YES | NO | Medium |
| Zecin-Deren, 2019 [110] | YES | NO | NO | YES | YES | NO | YES | NO | Medium |
| Zecin-Deren, 2019 [110] | YES | NO | NO | YES | NO | NO | YES | NO | High |
